# Computer-Aided Diagnosis in Multiparametric MRI of the Prostate: An Open-Access Online Tool for Lesion Classification with High Accuracy

**DOI:** 10.3390/cancers12092366

**Published:** 2020-08-21

**Authors:** Stephan Ellmann, Michael Schlicht, Matthias Dietzel, Rolf Janka, Matthias Hammon, Marc Saake, Thomas Ganslandt, Arndt Hartmann, Frank Kunath, Bernd Wullich, Michael Uder, Tobias Bäuerle

**Affiliations:** 1 Department of Radiology, University Hospital Erlangen, 91054 Erlangen, Germany; michi.schlicht@googlemail.com (M.S.); dietzelmatthias2@hotmail.com (M.D.); rolf.janka@uk-erlangen.de (R.J.); matthias.hammon@gmail.com (M.H.); marc.saake@uk-erlangen.de (M.S.); michael.uder@uk-erlangen.de (M.U.); Tobias.Baeuerle@uk-erlangen.de (T.B.); 2 Department of Medical Informatics, Friedrich-Alexander-University Erlangen-Nuremberg, 91058 Erlangen, Germany; thomas.ganslandt@medma.uni-heidelberg.de; 3 Heinrich-Lanz-Center for Digital Health, Department of Biomedical Informatics, University Medicine Mannheim, Heidelberg University, 68177 Mannheim, Germany; 4 Institute of Pathology, University Hospital Erlangen, 91054 Erlangen, Germany; arndt.hartmann@uk-erlangen.de; 5 Department of Urology and Paediatric Urology, University Hospital Erlangen, 91054 Erlangen, Germany; frank.kunath@uk-erlangen.de (F.K.); bernd.wullich@uk-erlangen.de (B.W.)

**Keywords:** artificial intelligence, computer-aided diagnosis, machine learning, prostate mpMRI, prostate cancer

## Abstract

Computer-aided diagnosis (CADx) approaches could help to objectify reporting on prostate mpMRI, but their use in many cases is hampered due to common-built algorithms that are not publicly available. The aim of this study was to develop an open-access CADx algorithm with high accuracy for classification of suspicious lesions in mpMRI of the prostate. This retrospective study was approved by the local ethics commission, with waiver of informed consent. A total of 124 patients with 195 reported lesions were included. All patients received mpMRI of the prostate between 2014 and 2017, and transrectal ultrasound (TRUS)-guided and targeted biopsy within a time period of 30 days. Histopathology of the biopsy cores served as a standard of reference. Acquired imaging parameters included the size of the lesion, signal intensity (T2w images), diffusion restriction, prostate volume, and several dynamic parameters along with the clinical parameters patient age and serum PSA level. Inter-reader agreement of the imaging parameters was assessed by calculating intraclass correlation coefficients. The dataset was stratified into a train set and test set (156 and 39 lesions in 100 and 24 patients, respectively). Using the above parameters, a CADx based on an Extreme Gradient Boosting algorithm was developed on the train set, and tested on the test set. Performance optimization was focused on maximizing the area under the Receiver Operating Characteristic curve (ROC_AUC_). The algorithm was made publicly available on the internet. The CADx reached an ROC_AUC_ of 0.908 during training, and 0.913 during testing (*p* = 0.93). Additionally, established rule-in and rule-out criteria allowed classifying 35.8% of the malignant and 49.4% of the benign lesions with error rates of <2%. All imaging parameters featured excellent inter-reader agreement. This study presents an open-access CADx for classification of suspicious lesions in mpMRI of the prostate with high accuracy. Applying the provided rule-in and rule-out criteria might facilitate to further stratify the management of patients at risk.

## 1. Introduction

Prostate cancer (PCa) is the second most frequently diagnosed cancer in men worldwide [1]. Over recent years, multiparametric magnetic resonance imaging (mpMRI) has become a pivotal tool in PCa management, including detection, staging, and treatment planning [2]. Updated year 2019 guidelines from the European Association of Urology (EAU) even endorse mpMRI prior to initial biopsy [3]. However, in daily clinical practice, a prostate mpMRI is still often performed only after several negative biopsies [4]. Thus, mpMRI has not yet progressed to a first-line modality, partially because of the substantial expertise required for reporting [4] and the use of an adequate acquisition technique.

To standardize the evaluation of prostate mpMRI, the Prostate Imaging Reporting and Data System (PI-RADS) was developed (current version 2.1, American College of Radiology, Reston, VA, USA) [5,6]. Two meta-analyses on PI-RADS v2 reported pooled sensitivities of 85% and 89% and specificities of 71% and 73% [7,8]. Although offering a highly systematic approach to evaluation, the inter-observer agreement for PI-RADS v2 remains moderate, even among experienced readers [9].

Computer-aided diagnosis (CADx) approaches have been proposed to assist physicians and provide either probability maps of cancer or malignancy scores for any region of interest (ROI) [10,11]. Many of these algorithms rely on proprietary, custom-built algorithms, which hampers their application in workflows outside the institutions where they have been established.

The aim of this study was to develop an open-access CADx tool for the classification of suspicious lesions detected in prostate mpMRI that offers high accuracy and an excellent inter-observer agreement of its input parameters.

## 2. Results

### 2.1. Patient Characteristics

**Figure 1:**
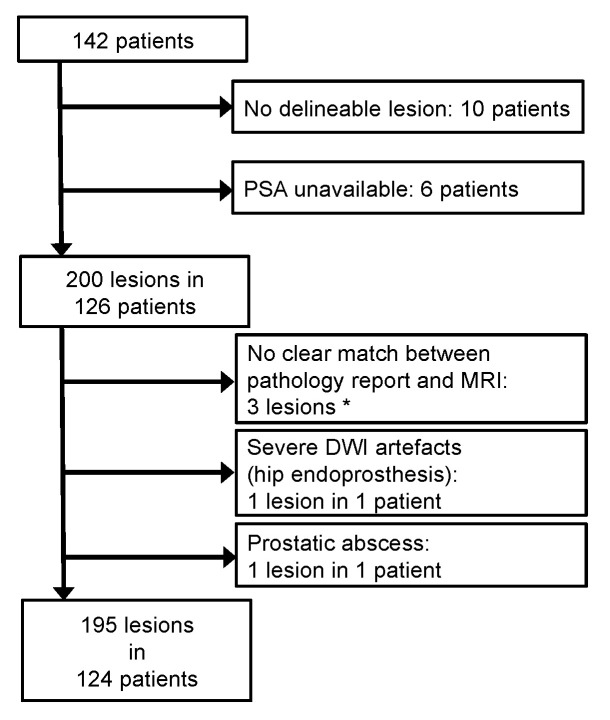
Patient flow chart. An initial database search retrieved 142 patients eligible for this retrospective study. However, 18 patients were excluded based on the exclusion criteria. In total, 124 patients with 195 lesions were included. (*) Three lesions could not be unambiguously matched between the pathology report and the MRI assessment. The affected patients, however, featured other lesions that were clearly matched.

Of 142 eligible patients, 18 were excluded based on the exclusion criteria, leaving 124 patients, with 195 lesions, being included in this study (Figure 1). Of 195 lesions, 83 were malignant (42.6%) and 54 of these malignant lesions represented peripheral zone PCa (65.1%). Of the 124 patients, 64 had one or more PCa lesion (51.6%). The mean patient age was 66 years (range, 46–84). Patients featured up to four reported prostate lesions (range, 1–4; median, 1).

### 2.2. Descriptive Statistics

**Figure 2:**
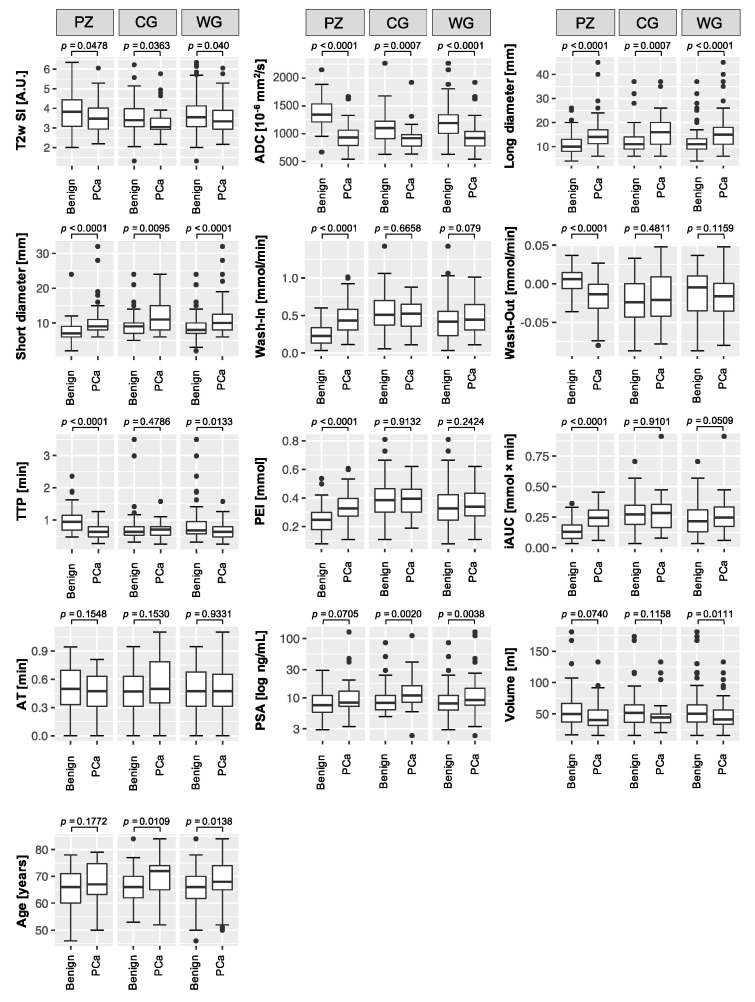
Boxplots of the acquired parameters. T2w signal intensity (SI), apparent diffusion coefficient (ADC), long and short diameter, dynamic contrast enhancement wash in and wash out, time to peak (TTP), peak enhancement intensity (PEI), initial area under the curve (iAUC), and arrival time (AT), and the additional parameters: prostate specific antigen (PSA) serum level, prostate volume, and patient age. Boxplots follow Tukey’s definition, with whiskers depicting the 1.5× interquartile ranges, and outliers marked as circles. The plots compare benign lesions (left boxes) with prostate cancer (PCa) lesions (right boxes), further divided into analyses of the whole gland (WG), and sub-analyses of the peripheral zone (PZ) and the central gland (CG). *p*-values, derived from Mann–Whitney *U* tests, are given above the plots.

PCa lesions featured significantly decreased T2w signal intensity (SI) and apparent diffusion coefficients (ADC), and significantly higher long and short diameters. In a sub-analysis of just the peripheral zone lesions, wash in, wash out, peak-enhancement-intensity (PEI), and the initial area under the curve (iAUC) were significantly different between malignant and benign lesions. Arrival time (AT) did not differ between malignant and benign lesions. Prostate-specific antigen (PSA) levels were significantly higher in PCa of the central gland, or when evaluating the whole gland, but not in a sub-analysis of peripheral zone PCa. Prostates with cancer were significantly smaller than prostates with benign lesions, but only on a whole-gland basis. PCa patients were significantly older, but not in a sub-analysis of just the peripheral zone (Figure 2).

### 2.3. CADx Development

The entire dataset was split into a training set and testing set. The testing set contained 20.0% of the lesions from 19.4% of the patients. There were no significant differences between the training and testing sets for any of the (semi-)quantitative parameters (all, *p* ≥ 0.24; Table 1). 

During feature selection on the training set, AT was the only parameter that did not contribute to increased accuracy, so was dismissed. The algorithm was trained with the retained parameters, listed in descending order regarding their variable importance: ADC, long diameter, PSA, T2w SI, age, prostate volume, iAUC, short diameter, PEI, wash in, wash out, TTP, and location (peripheral zone vs. central gland). To assess inter-reader agreement, the testing set lesions were independently re-assessed by R2, with excellent agreement (all, ICC ≥ 0.87; Table 2).

### 2.4. CADx Performance

**Figure 3:**
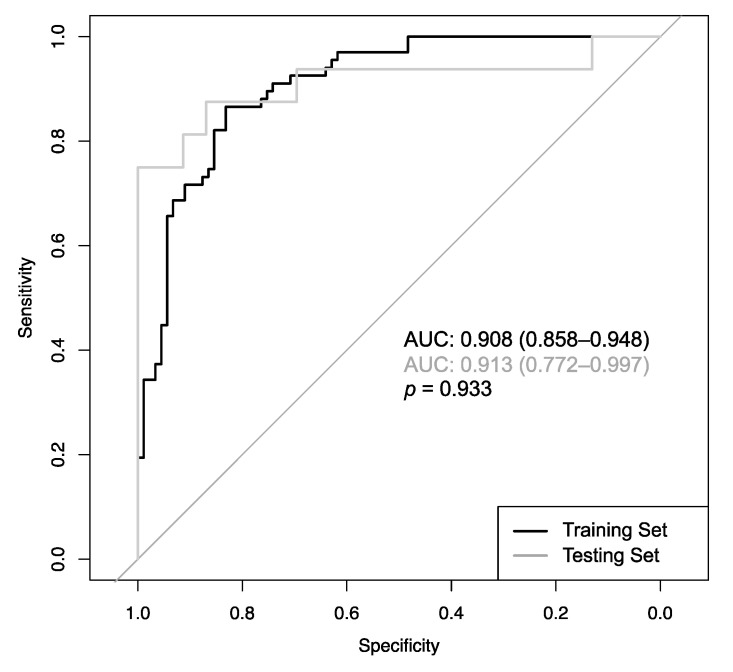
Receiver Operating Characteristic curves of the CADx model determined from the 10-fold cross-validation (CV) procedure during training (black line; AUC = 0.908) and testing (grey line; AUC = 0.913), with no significant difference (*p* = 0.933).

The CADx featured an area under the Receiver Operating Characteristic curve (AUC_ROC_) of 0.908 (CI: 0.858–0.948) during training and 0.913 (CI: 0.772–0.997) during testing, with no significant difference (*p* = 0.933; Figure 3). No other accuracy parameters, including sensitivity, specificity, PPV and NPV, overall accuracy, and positive/negative likelihood ratio differed significantly (Table 3).

The CADx also calculates a probability score, so that every point on the ROC curve in Figure 3 resembles a distinct cut-off with a distinct sensitivity and specificity. These cut-offs can be used to classify any given lesion with specific error rates. Regarding the lesions from the training set, 35.8% of the cancerous lesions were classifiable with a maximum error rate of 2%, and 49.4% of the benign lesion were classifiable with a maximum error rate of 2%. An overview of error rates from 1 to 5% and the number and percentages of lesions classifiable with these specific rates are provided in Table 4.

**Figure 4:**
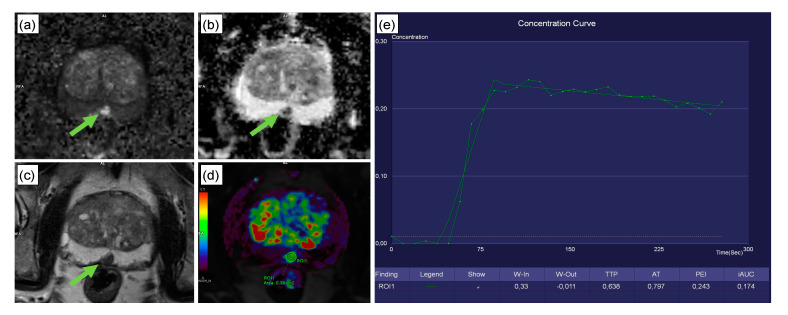
Clinical case from the testing set. This 74-year-old patient was referred to radiology for prostate MRI because of an increase in serum PSA levels to 8 ng/mL and the suspicion of prostate cancer. Multiparametric MRI revealed a 10 × 7 mm PI-RADS 4 lesion dorsally in the mid/apical peripheral zone (green arrow; classification according to PI-RADS v2.1 [6]). The lesion featured high signal intensity in DWI (**a**), along with a strong diffusion restriction in ADC (**b**). T2w axial images showed a signal decrease in the sharply delineated lesion (**c**). The lesion featured a steep wash-in curve, along with a decent wash out (**d,e**). Subsequent histopathology revealed scarred prostate tissue and few prostate glands with urothelial metaplasia, but no evidence of malignancy. The CADx correctly excluded malignancy for this peripheral zone PI-RADS 4 lesion, with a false negative rate of 3%. The patient received a follow-up examination 9 months later in which the lesion had remained unchanged.

**Figure 5:**
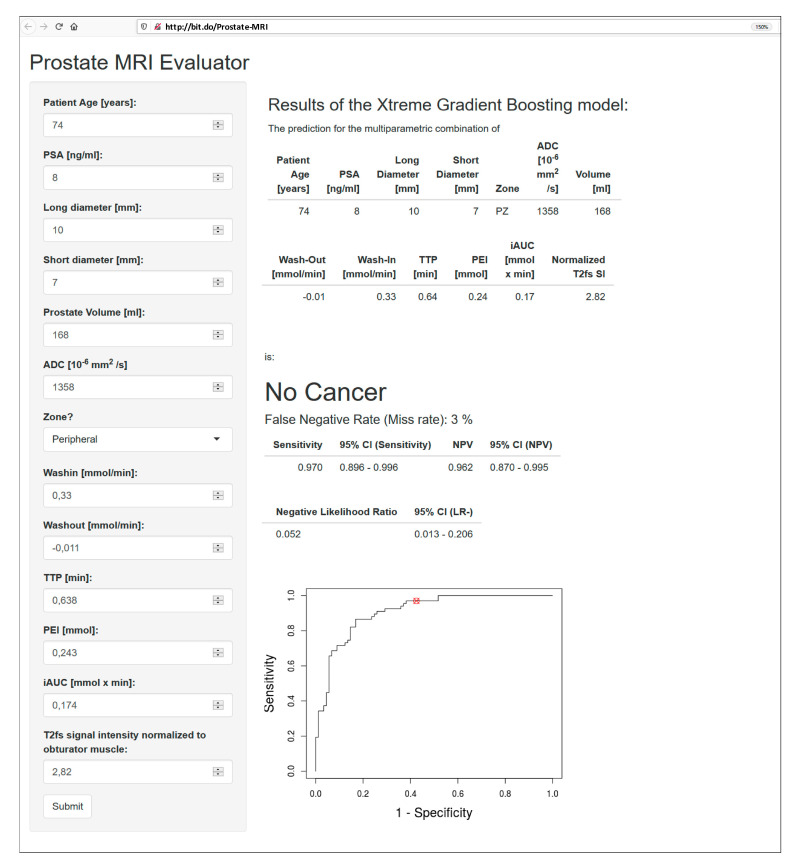
Interface of the open-access web application, accessible at http://bit.do/Prostate-MRI, with an analysis of the peripheral zone PI-RADS 4 lesion presented in Figure 4. The lesion was correctly classified as benign, with an error rate of 3%. Its location on the ROC curve is highlighted with a red circle. Sensitivity: 97.0% (95% CI: 89.6–99.6%); NPV: 96.2% (95% CI: 87.0–99.5%); negative likelihood ratio: 0.052 (95% CI: 0.013–0.206).

Figure 4 presents a clinical case with a PI-RADS 4 lesion dorsally in the mid-to-apical peripheral zone. Histopathology revealed scarred prostate tissue and few prostate glands with urothelial metaplasia, but no evidence of malignancy. The CADx classified this lesion correctly as benign. Figure 5 depicts the interface of the web application when analyzing the peripheral zone lesion presented in Figure 4.

## 3. Discussion

This study presents a classification CADx to discriminate between malignant and benign lesions in prostate mpMRI using an open-access web application. The CADx features an ROC_AUC_ of 0.908, which is within the upper range of previously published CADx systems [11,12] that ranged from 71% [13] to 97% [14] for 1.5 T scanners, and from 77% [15] to 95% [16] for 3 T scanners. Nevertheless, comparing diagnostic accuracies of CADx systems directly is almost impossible, since different studies were performed with different magnetic field strengths, were based on mono-, bi-, or multiparametric image material, defined different variables as a target to be optimized (e.g., overall accuracy, sensitivity, specificity, and ROC_AUC_), or were based on relatively low patient numbers. The threshold value for the diagnosis of malignancy of the algorithm presented here was calculated from multiparametric images from 1.5 and 3 T scanners in such a way that the ROC_AUC_ was maximized.

PI-RADS v2 is a relatively new system that was introduced in 2015. Since then, several studies have assessed its diagnostic accuracy; One meta-analysis, consisting of 13 studies, revealed a pooled sensitivity of 85% and specificity of 71% for PCa detection [8]; A second meta-analysis included 21 studies and reported pooled sensitivities and specificities of 89% and 73%, respectively [7]. PI-RADS v2.1, however, is expected to improve diagnostic accuracy especially for lesions of the transition zone [17]. In our cohort, a sensitivity of 82.1% and specificity of 85.4% was achieved. 

Several multireader studies have also investigated the inter-reader agreement of PI-RADS v2 [9,18]. Interestingly, even studies among experts describe only moderate agreement (kappa = 0.55) [9], which might be due to the subjective image impressions used for the PI-RADS classifications. PI-RADS v2.1 was developed, among other objectives, with the anticipation of an increased interreader agreement, which has recently been shown for lesions of the transition zone [17,19]. Nevertheless, PI-RADS v2.1-based evaluation is still subject to perceived image impressions, which makes it possibly susceptible to interobserver variability. (Semi-)quantitative parameters have the potential to improve agreement, and the inter-reader agreement of all parameters in this study was excellent. Another strength of this study is the possibility to rule-in or rule-out malignancy with defined error rates, which could help to stratify patients for either upfront biopsy or short-term follow up, at least in selected cases. However, these retrospective results should be handled with care, and the CADx should not be used for comprehensive assessments before validation in prospective studies.

This study has several limitations. First, the standard of reference (SOR) was histopathology, based on MRI/ultrasound fusion-guided biopsies combined with systematic biopsies. This combined approach offers a sensitivity of 85% [20]. It is, therefore, possible that some lesions used for training the algorithm were inadvertently regarded as “benign”, but actually represented an unrecognized PCa. One solution to this would have been to rely on radical prostatectomies. However, this would have drastically reduced the number of patients available for this study. To date, a combination of systematic with targeted biopsies is still considered to be the gold standard for PCa confirmation [21]. Therefore, choosing it as an SOR seemed a reasonable approach.

Second, our definition of PCa included Gleason-6-lesions, which are commonly considered clinically non-significant, whereas the definition of clinically significant PCa remains an ongoing debate [5,22]. Defining lesions with a Gleason Score ≥6 as PCa seems justifiable against the background of a need for biopsy also for PCa of questionable clinical significance.

Third, training of the CADx algorithm was performed on 156 lesions, while testing was performed on 39 lesions. The diagnostic measures in the web application were calculated from the cross-validation performed during training. This approach is not uncommon, and several studies state accuracy measures solely based on cross-validation [4,23,24,25]. Although our study’s accuracies were not significantly different between the training and testing sets, it would be desirable to have the final accuracy measures calculated from an independent dataset. However, the 39 lesions from the testing set were too few to provide an adequate data basis for diagnostic measures. Future studies should be conducted to independently validate the results obtained in this study. The open-access online form will facilitate those studies and even allow multicentre studies.

Fourth, dynamic contrast-enhance (DCE) MRI assessment is challenging because multiple techniques for calibration and modelling exist [26] and the conversion of MRI signal intensities to contrast agent concentrations is not a trivial problem. The dynamic parameters used in this study, however, were acquired using a widely available workstation and showed excellent reproducibility. Nevertheless, the dependence on a commercial software solution is a weakness that could prevent a larger-scale application of the algorithm presented here. Future extensions of our model will, on the one hand, aim to include parameters extractable with different additional DICOM viewers, including open-source solutions for DCE evaluation, and, on the other hand, adapt the algorithm to also support bi-parametric MRI for analyses of unenhanced prostate MRIs.

## 4. Materials and Methods 

### 4.1. Patients

This retrospective study complies with the declaration of Helsinki. The institutional review board of the Friedrich-Alexander-Universität Erlangen-Nürnberg (260_19 Bc, 2019/08/30), Germany, approved this study, with waiver of informed consent. We performed a database search for patients who received prostate mpMRI between 06/2014 and 07/2017, and had a combined MRI/ultrasound targeted and systematic transrectal ultrasound (TRUS)-guided biopsy within one month after the mpMRI. Histopathology of the biopsy cores served as an SOR.

Exclusion criteria included no delineable lesion in MRI, unavailable PSA serum levels, or severe imaging artefacts. Lesions were excluded if there was no clear match between imaging and pathology report, or when they were not covered by the PI-RADS reporting scheme (e.g., abscesses).

### 4.2. Imaging

#### 4.2.1. Imaging Parameters

The MRI protocol was optimized following international recommendations and current practice [5,27]; the patient was in supine position using either 1.5 T or 3 T scanners (Avanto/Aera; Verio/Skyra; Siemens Healthineers, Erlangen, Germany) and a pelvic-phased-array coil. Protocols included T2-weighted scans with spectral fat-saturation, DCE sequences, T1-weighted scans with and without contrast-enhancement, and diffusion-weighted imaging (DWI; Table 5). The contrast medium (0.1 mmol/kg body weight gadobutrol, Bayer Schering Pharma, Berlin, Germany) was injected into an antecubital vein prior to the DCE acquisition, with a flow rate of 2.0 mL/s, followed by a 20 mL saline flush. 

#### 4.2.2. Image Assessment

**Figure 6:**
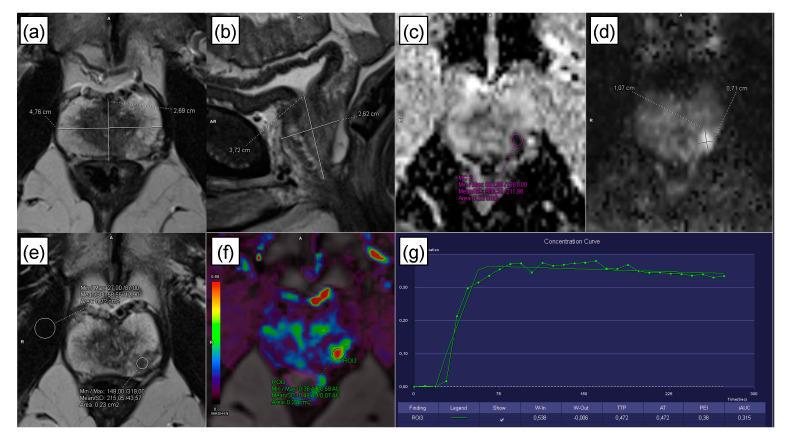
Image assessment. (**a**,**b**) Prostate volume was calculated from the diameters measured in axial and sagittal T2w images, as described in the PI-RADS v2.1 guidelines [6]. A circular region of interest (ROI) was placed within a lesion, for lesions of the peripheral zone, preferably on the ADC map (**c**) in additional consideration of DWI (calculated b-value of 1500 s/mm^2^; (**d**)), and for lesions of the transitional zone/central gland, preferably in the axial T2w sequence (**e**) [5,6]. Following placement, the ROI was copied to the other sequences using the software. If lesion measurement was difficult or compromised on ADC (for peripheral zone lesions) or T2w (for transitional zone/central gland lesions), measurements were performed on the sequence that showed the lesion best [5,6], e.g., in the above example, lesion dimensions were assessed on DWI (d). (**f**,**g**) Time-intensity curves were automatically calculated by the software, yielding the dynamic parameters: wash in, wash out, time to peak (TTP), peak enhancement intensity (PEI), arrival time (AT), and initial area under the curve (iAUC).

All mpMRI were assessed by a board-certified radiologist (R1, S.E., six years of experience in prostate MRI, >400 reads/year) using a clinical post-processing platform (syngo.via VB30A, Siemens Healthineers, Erlangen, Germany). Blinded to the SOR, R1 measured the maximum (“long”) diameter in axial orientation and the perpendicular (“short”) diameter of each lesion and defined a circular ROI within the lesion, which served as a mask that was copied to the other sequences. Hereby, syngo.via offers automatic image registration to ensure congruent positioning of ROIs in all sequences. The following measurements were acquired:

- Prostate volume, calculated according to [6] (Figure 6a,b). Prostate volume positively correlates with PSA level [28], and calculating PSA density can improve the diagnosis of clinically significant PCa [29]; therefore, including prostate volume as a parameter seemed reasonable.
- ADC (10^–6^ mm^2^/s; Figure 6c,d), because PCa aggressiveness and ADC correlate inversely [30].
- Normalized T2w SI, with the SI of the lesion divided by the SI of the internal obturator muscle. T2w SI is known to be particularly valuable for the evaluation of transitional zone lesions [30] (Figure 6e).
- The 2-dimensional lesion size in axial orientation (Figure 6e). Size is a recognized criterion used to distinguish PI-RADS 4 from PI-RADS 5 lesions [6].

DCE-MRI is an established mpMRI component. PI-RADS v2 and v2.1, however, restricted its application to cases with indeterminate DWI in peripheral zone lesions, which was justified by the lack of expert consensus and difficulties in interpreting DCE by eye. A (semi-)quantitative assessment, however, could improve consistency. Therefore, the following DCE parameters were assessed using syngo.via’s Tissue 4D workflow:

- TTP (time from bolus arrival to end of wash in),
- AT (start of contrast enhancement),
- Wash in (slope of the line between bolus arrival and end of wash in),
- Wash out (slope of the line between start of wash out and end of measurement),
- PEI, and
- iAUC (in 60 s).

Syngo.via’s Tissue 4D workflow hereby converts MR signal intensities to Gadolinium concentrations as described in [31], i.e., from the relative enhancement, TR, flip angle, estimated tissue T1, and contrast agent relaxivity.

Assessment of lesions was performed according to the current version of PI-RADS (version 2.1) [6].

Figure 6 further explains the parameter acquisition. In addition, patient age and PSA value were noted and included as potential predictive features.

A representative subset of the lesions (*n* = 39) was re-assessed by a second reader (R2, T.B., 10 years of experience, >400 reads/year), who collected the quantitative parameters again to determine inter-observer variability. R2’s measurements were not used to train the algorithm. 

### 4.3. Histopathology

All patients underwent a combination of a TRUS-guided 12-core systematic biopsy with an MRI/ultrasound fusion-guided biopsy (up to 2 additional cores per lesion) [21], performed by urologists with >10 years of experience (Frank Kunath and Bernd Wullich, both from the Department of Urology and Paediatric Urology, University Hospital Erlangen, 91054 Erlangen, Germany). Cores were individually labelled according to their location and analysed by a uro-pathologist with >15 years of experience (Arndt Hartmann, Institute of Pathology, University Hospital Erlangen, 91054 Erlangen, Germany). 

### 4.4. CADx Development, Statistics, and Open-Access Internet Application

#### 4.4.1. CADx Development

The prediction of malignancy in any given prostate lesion was regarded as a classification problem to be solved by an Extreme Gradient Boosting (XGBoost, University of Washington, Seattle, WA, USA) algorithm. XGBoost creates a prediction model as an ensemble of weak classifiers, building many decision trees and integrating them into one cumulative prediction model to obtain a performance that exceeds any of the constituent classifiers. The model was calculated in RStudio 3.4.1 (RStudio, Inc., Boston, MA, USA), using caret 6.0-81 [32] and xgboost 0.71.2 [33].

To assess the algorithm’s performance, patients were split into a training set (*n* = 156 lesions in *n* = 100 patients) and a testing set (*n* = 39 lesions in *n* = 24 patients). As several patients featured more than one lesion, to avoid data leakage, care was taken that all lesions from one patient examination were either assigned to the training or to the testing set.

Feature selection was performed exclusively on the training set by calculating the variable importance from an untuned random forest—this algorithm is directly implemented within the R caret package [32] and offers the possibility to quantify the importance of particular features when used in tree-based classification algorithms. Features that did not improve the overall accuracy were dismissed. CADx optimization was focused on maximizing the area under the curve of the Receiver Operating Characteristic (AUC_ROC_). To determine the optimal hyperparameter combination, a grid search among 100,000 random combinations was performed (Table A1). To ensure generalizability, a 10-fold cross-validation approach was chosen. The final algorithm was tested on the testing set. 

#### 4.4.2. Statistics

Statistical analyses were performed using RStudio 3.4.1 (RStudio, Inc., Boston, MA, USA). Mann–Whitney *U* and exact Fisher or χ^2^ tests were applied for comparisons of continuous and categorical variables, respectively. ROC curves were compared using DeLong’s test. Inter-observer agreement was determined using the intraclass correlation coefficient (ICC), with ICC > 0.75 regarded as “excellent” [34]. *p*-Values < 0.05 were considered statistically significant. Confidence intervals (CI) were calculated at 95%.

AUC_ROC_ was used to estimate diagnostic accuracy. Further analyses included contingency tables to assess sensitivity, specificity, positive and negative predictive value (PPV and NPV, respectively), and positive and negative likelihood ratio, along with their respective 95% CI. 

In addition, potential decision rules were evaluated; such decision rules have been described as promising tools for other cancer types [35,36], enabling diagnostic statements of either presence (rule in) or absence (rule out) of malignancy. As a common principle, if sensitivity is high, a “negative” test rules out malignancy, and if specificity is high, a “positive” test rules in malignancy [37]. Therefore, rule-in and rule-out criteria were defined as follows:

Rule-out criteria were present if the CADx excluded PCa with an “error rate” (false-negative rate, FNR) of 1–5%. Likewise, rule-in criteria were explored at false-positive rates (FPR) of 1–5%.

#### 4.4.3. Open-Access Internet Application

The CADx algorithm was implemented into an open-access internet application with Shiny [38]. For any given lesion, this application provides a diagnosis based on the parameters provided. The diagnostic accuracy is further specified by the corresponding “error rate”, PPV/NPV, and the respective likelihood ratio.

## 5. Conclusions

This study presents an open-access CADx for the classification of suspicious lesions in prostate mpMRI, with high accuracy and excellent inter-reader agreement. Applying the rule-in and rule-out criteria could help to further stratify at-risk patients.

## Figures and Tables

**Table 1:** Comparison of the acquired parameters and additional lesion characteristics between the lesions from the training set (*n* = 156), and the testing set (*n* = 39).

Parameter	Training Set		Testing Set		*p*-Value
	Median	Range	Median	Range	
Patient age [years]	67	46–84	66	53–79	0.79
Prostate volume [mL]	46.0	15.6–180.8	41.8	22.5–173.5	0.29
PSA [ng/mL]	8.46	2.91–129	8.00	2.3–40	0.38
T2w SI [A.U.]	3.39	1.31–6.22	3.61	2.65–6.34	0.24
ADC [10^–6^ mm^2^/s]	1032.5	542–2267	1067	621–2151	0.76
Long diameter [mm]	12	4–45	13	4–28	0.51
Short diameter [mm]	9	4–32	8	2–24	0.60
Wash in [mmol/min]	0.419	0.032–1.423	0.483	0.07–0.926	0.51
Wash out [mmol/min]	−0.009	−0.087–0.048	−0.017	−0.059–0.021	0.29
TTP [min]	0.653	0.249–3.496	0.638	0.452–1.257	0.85
AT [min]	0.472	0–1.1	0.384	0–0.943	0.81
PEI [mmol]	0.326	0.079–0.809	0.360	0.109–0.598	0.45
iAUC [mmol·min]	0.230	0.035–0.912	0.261	0.04–0.472	0.68
Gleason Score (PCa only)	7	6–9	7	6–9	0.89
Histopathology	PCa: *n* = 67; Benign: *n* = 89		PCa: *n* = 16; Benign: *n* = 23		0.86
Zone	PZ: *n* = 76; CG: *n* = 80		PZ: *n* = 21; CG: *n* = 18		0.60

The table provides the median and the range for each of the numerical variables, along with Mann–Whitney *U* test *p*-values. For Gleason Scores, the median, range and *p*-values from a χ^2^ test are given; for the categorical variables (peripheral zone/central gland; PZ and CG, respectively, and cancerous vs. benign lesions), exact Fisher’s test *p*-values are given.

**Table 2:** Intraclass correlation coefficients (ICCs) of the imaging parameters.

Parameter	ICC	95% CI
T2w SI	0.88	0.78–0.93
ADC	0.87	0.72–0.94
Long diameter	0.87	0.76–0.93
Short diameter	0.90	0.80–0.95
Wash in	0.88	0.78–0.93
Wash out	0.93	0.88–0.96
TTP	0.93	0.88–0.96
PEI	0.88	0.79–0.94
iAUC	0.90	0.81–0.94
Volume	0.93	0.84–0.97

ICCs were used to determine inter-reader agreement between R1 and R2, and are provided along with their 95% confidence intervals (CI). All parameters used in the CADx model showed excellent agreement.

**Table 3:** Comparison of diagnostic accuracy measures of the CADx model performance during training and testing.

Parameter	Training Set		Testing Set		*p*-Value
	Estimation	95% CI	Estimation	95% CI	
Sensitivity	82.1%	0.708–0.904%	81.2%	54.4–96.0%	0.937
Specificity	85.4%	0.763–0.92%	82.6%	61.2–95.0%	0.759
PPV	80.9%	0.695–0.894%	76.5%	50.1–93.2%	0.703
NPV	86.4%	0.774–0.928%	86.4%	65.1–97.1%	1.000
Accuracy	84.0%	0.773–0.894%	82.1%	66.5–92.5%	0.787
Positive Likelihood Ratio	5.62	3.36–9.40	4.67	1.86–11.7	0.776
Negative Likelihood Ratio	0.21	0.13–0.35	0.23	0.08–0.64	0.901

The table lists sensitivity, specificity, positive and negative predictive values (PPV and NPV, respectively), overall classification accuracy, and positive and negative likelihood ratios, along with their 95% confidence intervals (CI). No significant differences were observed (all, *p* ≥ 0.703).

**Table 4:** Numbers (percentages) of lesions classifiable with defined error rates.

Error Rate	Rule In	Rule Out
<1%	13/67 (19.4%)	43/89 (48.3%)
<2%	24/67 (35.8%)	44/89 (49.4%)
<3%	24/67 (35.8%)	56/89 (62.9%)
<4%	28/67 (41.8%)	58/89 (65.2%)
<5%	34/67 (50.7%)	60/89 (67.4%)

The table lists the numbers and percentages of lesions classifiable by the CADx with error rate thresholds of 1–5%.

**Table 5:** MRI acquisition parameters.

Parameter	1.5 T				3 T			
	T2w TSE	DCE (VIBE)	T1w TSE	DWI	T2w TSE	DCE (VIBE)	T1w TSE	DWI
TR [ms]	7440	4.2	433	5300	4000	5.04	688	5090
TE [ms]	101	1.58	10	70	101	1.7	12	57
ETL	23	1	3		25	1	3	
Flip angle [°]	160	12	154	18	150	15	140	180
Field of view [mm^2^]	200 × 200	259 × 259	200 × 200	200 × 200	200 × 200	200 × 200	308 × 380	200 × 200
Matrix	320 × 275	192 × 154	256 × 192	112 × 112	320 × 310	128 × 102	333 × 512	94 × 118
Slice thickness [mm]	3	3.5	3	3.5	3	3	5	3.5
Number of slices	28	22	28	20	26	24	40	25
Averages	3	1	1	1	3	1	1	1
Duration [min:s]	04:20	04:21	02:45	07:06	03:52	04:34	03:29	06:03

Acquisition parameters are given for both 1.5 T and 3 T MRI for the sequences: T2w turbo spin echo (TSE), dynamic contrast enhancement volume interpolated breathhold examination (DCE VIBE), T1w TSE, and diffusion-weighted imaging (DWI; b-values: 0; 100; 800, and a calculated b-value of 1500 s/mm^2^), along with their repetition times (TR), echo times (TE), echo train lengths (ETL), flip angles, fields of view, matrix sizes, slice thicknesses, the numbers of acquired slices, averages, and sequence durations.

## Appendix A

**Table A1:** Applied hyperparameter settings of the Extreme Gradient Boosting model.

Parameter	Value
subsample	0.63
nrounds	121
eta	0.34
gamma	0.2
max_depth	8
min_child_weight	1.3
colsample_bytree	0.76
rate_drop	0.49
skip_drop	0.88

The optimal hyperparameter combination was determined in a grid search. It is important to mention that all hyperparameters act in harmonic orchestration: The XGBoost algorithm fits a boosted tree to a training dataset. Setting subsample to 0.63 means that XGBoost will randomly collect 63% of the data instances to train trees, and use the remaining 37% for validation. This approach helps to prevent overfitting. ROC_AUC_ are calculated for both the training and validation dataset and are retained. One subsample in the training dataset is then swapped with a validation subsample. Once again, the algorithm fits a boosted tree to the training data, calculates the evaluation scores, and so on. This process repeats until every subsample has served as a part of both the training and validation sets. Another boosted tree is added and the process is repeated. This continues until the total number of boosted trees fitted to the training data is equal to nrounds.

During the above processes, eta controls the learning rate, scaling the contribution of each tree by a factor of 0 < eta < 1 when it is added to the current approximation. Eta is needed to prevent overfitting by making the boosting process more conservative. Lower values of eta imply larger values of nrounds, and low eta values make a model more robust to overfitting, but slower to compute. Gamma controls the minimum loss reduction required to establish a further partition on a leaf node of the tree. The larger gamma, the more conservative the algorithm will be. Max_depth controls the maximum depth of each tree. Min_child_weight defines the minimum sum of instance weight needed in a child. If the tree partition step results in a leaf node with the sum of instance weight being less than min_child_weight, then the building process will discontinue partitioning. The larger min_child_weight, the more conservative the algorithm will be. Colsample_bytree refers to the fraction of randomly selected features used to construct each tree. Rate_drop defines the fraction of previous trees to drop, and skip_drop defines the probability of skipping the dropout procedure during a boosting iteration [33].
